# Semi-supervised prediction of protein subcellular localization using abstraction augmented Markov models

**DOI:** 10.1186/1471-2105-11-S8-S6

**Published:** 2010-10-26

**Authors:** Cornelia Caragea, Doina Caragea, Adrian Silvescu, Vasant Honavar

**Affiliations:** 1 Artificial Intelligence Research Laboratory, Department of Computer Science,Iowa State University, Ames, IA, 50010, USA; 2 Center for Computational Intelligence, Learning, and Discovery, Iowa State University, Ames, IA, 50010, USA; 3 Computer and Information Sciences, Kansas State University, Manhattan, KS, 65501, USA

## Abstract

**Background:**

Determination of protein subcellular localization plays an important role in understanding protein function. Knowledge of the subcellular localization is also essential for genome annotation and drug discovery. Supervised machine learning methods for predicting the localization of a protein in a cell rely on the availability of large amounts of labeled data. However, because of the high cost and effort involved in labeling the data, the amount of labeled data is quite small compared to the amount of unlabeled data. Hence, there is a growing interest in developing *semi-supervised methods* for predicting protein subcellular localization from large amounts of unlabeled data together with small amounts of labeled data.

**Results:**

In this paper, we present an Abstraction Augmented Markov Model (AAMM) based approach to semi-supervised protein subcellular localization prediction problem. We investigate the effectiveness of AAMMs in exploiting *unlabeled* data. We compare semi-supervised AAMMs with: (i) Markov models (MMs) (which do not take advantage of unlabeled data); (ii) an expectation maximization (EM); and (iii) a co-training based approaches to semi-supervised training of MMs (that make use of unlabeled data).

**Conclusions:**

The results of our experiments on three protein subcellular localization data sets show that semi-supervised AAMMs: (i) can effectively exploit unlabeled data; (ii) are more accurate than both the MMs and the EM based semi-supervised MMs; and (iii) are comparable in performance, and in some cases outperform, the co-training based semi-supervised MMs.

## Background

The problem of predicting subcellular protein localization is important in cell biology, because it can provide valuable information for predicting protein function and protein-protein interactions. Furthermore, the location of proteins in their designated subcellular compartments is essential for the proper functioning of the cell. Abnormal subcellular localization has been correlated with diseases such as cancer [1].

Many supervised machine learning methods have been successfully applied to the problem of predicting the subcellular localization of a protein, which can be formulated as a sequence classification problem [2], where the amino acid sequence of a protein is used to classify it in localization classes. For example, Park and Kanehisa [3] trained Support Vector Machine (SVM) classifiers using as features, frequencies of occurrence of pairs of amino acids, with 0 to 3 gaps between them. Emanuelsson et al. [4] developed a Neural Network-based approach using only information available in the N-terminal sequence. Höglund et al. [5] integrated information from the N-terminal sequence, amino acid composition, and protein sequence motifs in an SVM-based approach. Ong and Zien [6] trained multiclass SVMs and used an automated combination of protein motif kernels, with motifs of length up to 5 extracted from the whole sequence, and from different subsequences of it, i.e., the first 15 and 60 amino acids, and the last 15 amino acids of the sequence. Scott et al. [7] developed a Bayesian network that predicts the subcellular localization of a target protein using its features, e.g., InterPro motifs and the subcellular localization of its interacting partners. Yuan [8] trained *k^th^* order Markov chain models, with *k* ranging from 1 to 8, and used an approximation technique to estimate the probability of each element in a sequence given the *k* contiguous previous elements.

The accuracy of classifiers obtained using supervised learning algorithms depends in part on the quantity of labeled data that is available. Recent advances in sequencing technologies have resulted in an exponential increase in the rate at which DNA and protein sequence data are being acquired [9]. Because annotating the sequences with their subcellular localization requires costly experiments and manual curation effort, reliable annotations are available for only a small fraction of protein sequences. However, even the *unlabeled* data can provide valuable information, i.e., they contain information about the joint probability distribution over sequence elements. Consequently, there is a significant interest in *semi-supervised algorithms *[10] that can exploit large amounts of unlabeled data together with limited amounts of labeled data in training classifiers to predict protein subcellular localization.

Formally, the semi-supervised learning problem can be defined as follows: Given training data *D* = (*D_L_*, *D_u_*) of labeled and unlabeled examples, where , **x***_l_* ∈ **R^d^**, *y_l_* ∈ *Y*, and  x*_u_* ∈ **R^d^**, |*D_L_*| ≪ |*D_U_*|, respectively; a hypothesis space *H*; and a performance criterion *P*, a learning algorithm *L* outputs a classifier *h* ∈ *H* that optimizes *P*. If |*D_L_*| = 0, the problem reduces to unsupervised learning; if |*D_U_*| = 0, it reduces to supervised learning. The input x can represent sequences over a finite alphabet *X*, x ∈ X*. During classification, the task of the classifier *h* is to accurately assign a new example x *_test_* to a class label *y* ∈ *Y*.

Recently, a variety of methods for semi-supervised learning have been developed in the literature (see [11], [10] for reviews). Such methods have been successfully applied in many areas including text classification [12], [13], [14], natural language processing [15], [16], [17], image annotation [18], and more recently bioinformatics and computational biology, [19], [20], [21]. However, semi-supervised learning methods have not been widely applied to the subcellular localization prediction problem.

One notable exception is the work of Xu et al. (2009) [22]. The authors applied Co-Forest, which is an algorithm proposed by Li and Zhou [23], to exploit *unlabeled* data in order to improve predictive accuracy on the protein subcellular localization prediction task. Co-Forest extends the co-training approach of Blum and Mitchell [13] by using an ensemble of *N* classifiers, called Random Forest [24]. Note that the original co-training approach uses only two classifiers [13]. Co-Forest works as follows: let *H^N^* = (*h*_1_,…,*h_N_*} denote an ensemble of *N* classifiers. For each classifier *h_i_* ∈ *H^N^*, let  denote the *concomitant ensemble* of *h_i_*, where  is defined as the set of classifiers in *H^N^* except *h_i_*, i.e., . An ensemble *H^N^* of *N* random trees is initially trained on . At each subsequent iteration, for each classifier *h_i_* ∈ *H^N^*, its* concomitant ensemble* examines the *unlabeled* examples . An *unlabeled* example along with the label predicted by  is *added* to the newly labeled set of *h_i_*,  if the number of classifiers in  that predict a particular label exceeds a predefined threshold. The classifier *h_i_* is re-trained on . The process is repeated until no tree in the Random Forest changes from one iteration to another [23].

In this paper, we present a novel semi-supervised approach to the problem of predicting protein subcellular localization. Specifically, we use abstraction augmented Markov models (AAMMs), which are variants of Markov models, to incorporate information available in the *unlabeled* data. AAMMs model the dependency of each element in a sequence on *abstractions* of *k* preceding elements [25]. The abstractions are organized into an abstraction hierarchy that groups together *k*-grams that induce similar conditional probabilities of the next letter in the sequence. An AAMM corresponds to a generative model for sequence data expressed in terms of random variables whose values correspond to abstractions over *k*-grams, in addition to the MM random variables [25]. AAMMs provide a simple way to incorporate unlabeled data into the model: first, the abstraction hierarchy is constructed using the entire training set including the unlabeled data. Next, the labeled data is used to estimate the parameters of a set of AAMMs (one for each class) based on the resulting abstraction hierarchy.

Thus, in effect, AAMMs: (i) exploit the relatively large amount of unlabeled data to discover abstractions that transform the sequence data x and, hence, effectively reduce the number of parameters used to specify the probability *p*(x); and (ii) use the resulting representation to estimate the posterior probability *p*(*y|*x). Hence, we hypothesize that AAMMs are likely to yield more robust estimates of *p*(*y|*x) than MMs when the amount of labeled data is much smaller compared to the amount of unlabeled data.

To test our hypothesis on the protein subcellular localization prediction task, we compare AAMMs that use both *labeled* and *unlabeled* data with AAMMs that use only *labeled* data, with the standard MMs, which can not make use of *unlabeled* data, and also with MMs that can incorporate *unlabeled* data through an expectation maximization approach (EM-MM) and a co-training approach. The results of our experiments show that AAMMs can make effective use of *unlabeled* data and significantly outperform EM-MMs when the amount of *labeled* data are very small, and relatively large amounts of *unlabeled* data are readily available. Here, because of the small amounts of *labeled* data available for estimating parameters, the ability of AAMMs to minimize overfitting (through parameter smoothing) turns out to be especially useful. The results also show that AAMMs are competitive with, and in some cases significantly outperform two co-trained MMs on different views of the data.

## Experiments and results

We present results of experiments on three protein subcellular localization data sets: **psortNeg, plant,** and **non-plant** data sets (see Data sets Section for details).

### Experimental design

Our experiments on the protein subcellular localization prediction task are designed to explore the following questions: (i) How does the performance of semi-supervised AAMMs, which use both *labeled* and *unlabeled* data compare to that of MMs trained only on *labeled* data? (ii) How do AAMMs compare with MMs when both use *unlabeled* data? (iii) How effective are AAMMs at exploiting unlabeled data to improve classification accuracy when the amount of labeled data is limited? Specifically, how does the performance of an AAMM trained using both *labeled* and *unlabeled* data compare to that of an AAMM trained using only *labeled* data when both take advantage of *abstraction*? To answer the first and second questions, we compared AAMMs trained using an abstraction hierarchy constructed from both *labeled* and *unlabeled* data with the standard MMs, which can not make use of *unlabeled* data, and with MMs that can incorporate *unlabeled* data through an expectation maximization approach (EM) [26]. To answer the third question, we compared AAMMs trained using an abstraction hierarchy constructed from both *labeled* and *unlabeled* data with AAMMs trained using an abstraction hierarchy constructed only from *labeled* data.

In the first set of experiments, we trained semi-supervised AAMMs and supervised MMs for **psortNeg, plant,** and **non-plant** data sets. We ran experiments with 1%, 10%, and 25% of the training data being used as labeled examples, and the rest being treated as unlabeled examples (by ignoring the class). To obtain the subsets of 1%, 10%, and 25% of labeled examples, we sampled examples, using a uniform distribution, from the training set. Semi-supervised AAMMs are trained for values of *m* that range from 1 to *N*, where *m* is the cardinality of the set of abstractions *A_m_* used as “features” in the classification model, and *N* is the number of unique *k*-grams. We learned a single abstraction hierarchy (AH) from both *labeled and unlabeled training data* and used it to train an AAMM for each class (from the labeled sequences). An MM is trained on the same fraction of labeled data as its AAMM counterpart.

In the second set of experiments, we trained AAMMs, MMs, and EM-MMs for all three data sets. In the case of AAMMs, we trained classifiers for *m* = 1500 (*m* is set to 1500 because this partitioning of the set of *k*-grams produces classifiers that use substantially smaller number of “features” compared to MMs, i.e., ≈ 8000 *k*-grams, and at the same time, the model compression is not very stringent so as to lose important information in the data through *abstraction*)*.* We denote by AAMM(l+u) an AAMM trained using an AH constructed from both *labeled* and *unlabeled* data, and by AAMM(l) an AAMM trained using an AH constructed only from *labeled* data, when it is necessary to distinguish between AAMMs training procedures. EM-MMs are trained on the same fractions of labeled and unlabeled data as their AAMM(l+u) counterparts, and AAMM(l) and MMs are trained on the same fraction of labeled data as their AAMM(l+u) counterparts.

Here, we fixed the number of *unlabeled* examples and varied the number of *labeled* examples. Specifically, we performed experiments with 1%, 5%, 10%, 15%, 20%, 25%, 35%, and 50% of the training data being used as labeled examples, and 50% being treated as unlabeled examples (by ignoring the class label). Note that the unlabeled subset of the training data is the same across all the experiments; the labeled subset of the training data is successively augmented to increase the amount of labeled data that is provided to the learner.

In the third set of experiments, we compared AAMMs with EM-MMs using a fixed the number of *labeled* examples and a variable number of *unlabeled* examples. We performed experiments with (i) 1% of training data being treated as labeled, while 1%, 10%, 25%, 50%, 75%, 90%, and 99% being treated as unlabeled; (ii) 10% of training data being treated as labeled, while 1%, 10%, 25%, 50%, 75%, and 90% being treated as unlabeled; (iii) 25% of training data being treated as labeled, while 1%, 10%, 25%, 50%, and 75% being treated as unlabeled. As before, to obtain the subsets of labeled and unlabeled examples, we sampled using a uniform distribution, from the training set. In all experiments, the class distribution in each labeled subset is the same as that in the entire training set.

In the fourth set of experiments, we compare the semi-supervised AAMM with the co-training procedure as described in [13]. The co-training procedure can be used with any learning algorithms for training two classifiers *h*_1_ and *h*_2_. In this study, we used two Markov models. Inspired from the work of Ong and Zien [6], instead of considering features extracted from the entire protein sequence, we considered two views on different subsequences. Specifically, the first view corresponds to features extracted from the first 60 amino acids of each sequence, whereas the second view corresponds to features extracted from the last 15 amino acids of each sequence. We trained each Markov model on a different view. Furthermore, in this experiment, we trained AAMMs on the two subsequences of the first 60 and the last 15 amino acids of each sequence, rather than the entire sequence.

## Results

For all of the experiments, we report the average classification accuracy obtained in a 5-fold cross-validation experiment. All models are trained using 3-grams extracted from the data. For **psortNeg, plant,** and **non-plant** data sets the number of 3-grams is 7970, 7965, and 7999, respectively. Although the number of all unique *k*-grams is exponential in *k*, for large values of *k*, many of the *k*-grams may not appear in the data (consequently, the counts for such *k*-grams are zero). Note that the number of unique *k*-grams is bounded by the cardinality of the* multiset* of *k*-grams extracted from *D*.

We define the relative reduction in classification error between two classifiers to be the difference in error divided by the larger of the two error rates. To test the statistical significance of results, we used the 5-fold cross-validated paired *t* test for the difference in two classification accuracies [27]. The null hypothesis that the two learning algorithms *M*_1_ and *M*_2_ have the same accuracy on the same test set can be rejected if |*t*(*M*_1_, *M*_2_)| >*t*_4,0.975_ = 2.776 (*p* < 0.05). We abbreviate |*t*(*M*_1_, *M*_2_)| by |*t*| in what follows.

AAMMs can provide more accurate models compared to MMs on the protein subcellular localization prediction task when the amount of labeled data is small compared to that of unlabeled data.

**Figure 1:**
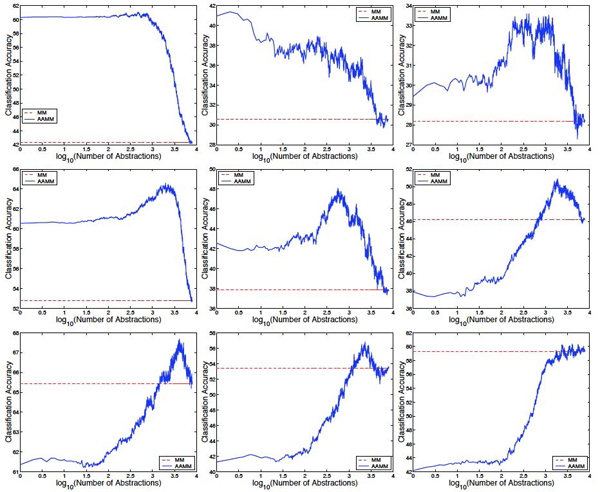
**Comparison of AAMMs with MMs**. Comparison of AAMMs with MMs for 1% (first row), 10% (second row), and 25% (third row) of labeled data available for **non-plant** (left), **plant** (center), and **psortNeg** (right), respectively.

Figure 1 shows results of the comparison of AAMMs with MMs for 1%, 10%, and 25% of labeled data, for **non-plant, plant**, and **psortNeg** data sets. Note that the *x* axis of all subfigures shows the number of abstractions *m* on a logarithmic scale. When only 1% and 10% of the training data are labeled (Figure 1, first and second rows), AAMMs significantly outperform MMs for many choices of m, on all three data sets. For example, on the 1% **plant** data set, with *m* = 200, the accuracy of AAMM is 38.72%, whereas that of MM is 30.53%, which represents 12% reduction in classification error, and |*t*| = 3.16 (the largest values of *t* are 27.58 for *m* = 4905, 21.91 for *m* = 2070, and 27.34 for *m* = 535 on **non-plant, plant**, and **psortNeg**, respectively). On the 10% plant data set, with *m* = 560, AAMM achieves an accuracy of 47.97%, compared to that of MM which is 37.87%, and |*t*| = 10.01.This represents 16% reduction in classification error. When we increased the fraction of labeled data to 25%, AAMMs still have a higher performance than MMs for many choices of m on **non-plant** and **plant** data sets, but become comparable in performance with MMs on **psortNeg** data set.

AAMMs trained using abstraction hierarchies constructed from both labeled and unlabeled protein subcellular localization data significantly outperform AAMMs trained using abstraction hierarchies constructed only from labeled protein subcellular localization data.

**Figure 2:**
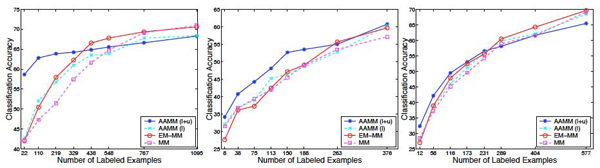
**Comparison of AAMM(l+u) with AAMM(l), MMs, and EM-MMs.** Comparison of AAMMs trained using an abstraction hierarchy learned from both *labeled* and* unlabeled* data, AAMM(l+u), with (i) AAMMs trained using an abstraction hierarchy learned only from *labeled* data, AAMM(l); (ii) Expectation-Maximization with Markov models, EM-MM; and (iii) Markov models, MM, on **non-plant** (left), **plant** (center), and **psortNeg** (right) data sets. *x* axis indicates the number of labeled examples in each data set corresponding to fractions of 1%, 5%, 10%, 15%, 20%, 25%, 35%, 50% of training data being treated as labeled data. The fraction of unlabeled data in each data set is fixed to 50%.

Figure 2 shows results of experiments that compare AAMM(l+u) with AAMM(l), MM, and EM-MM on **non-plant, plant**, and **psortNeg** data sets. The *x* axis indicates the number of labeled examples in each data set. The number of unlabeled examples is kept fixed and is equal to the rightmost number of labeled examples on the *x* axis of each plot.

As can be seen in the figure, AAMM(l+u) significantly outperforms AAMM(l) on all three data sets when small fractions of labeled data are available. For example, with 110 labeled sequences on **non-plant** (i.e., 5% of labeled data), AAMM(l+u) achieves 63% accuracy while AAMM(l) achieves 52%, which gives 23% reduction in classification error (|*t*| = 7.2). Strikingly, on the same data set, with only 22 labeled sequences (i.e., 1% of labeled data), AAMM(l+u) achieves 59% accuracy as compared to 43% obtained by AAMM(l), which gives 28% reduction in classification error (|*t*| = 9.73). Hence, AAMM(l+u) are able to incorporate information available in the unlabeled data (i.e., joint probability distributions of contiguous amino acids in a sequence) to learn more robust abstraction hierarchies than AAMM(l) when the labeled training set is limited in size (thereby, reducing the risk of *overfitting*).

Furthermore, AAMM(l+u) decreases the need for large numbers of labeled data. Specifically, on **non-plant**, AAMM(l+u) achieves 63% accuracy with 110 labeled examples, which is matched by that of AAMM(l) with 438 labeled examples (≈ 4 times more labeled data). However, when the fraction of labeled data is large, and hence, good estimates of model parameters can be obtained from such data, there is not much need for unlabeled data. For example, AAMM(l+u) becomes similar in performance with AAMM(l) on **non-plant** using 35% and 50% of labeled data (the null hypothesis is not rejected, |*t*| = 1.38 and |*t*| = 0.26, respectively).

As expected, the performance of AAMM(l+u) increases with the increase in the amount of labeled data. For example, on psortNeg with 12 labeled sequences (i.e., 1% of labeled data), AAMM(l+u) achieves 32% accuracy while AAMM(l+u) with 289 labeled sequences (i.e., 25% of labeled data) achieves 58% accuracy, which corresponds to 38% reduction in classification error.

AAMMs are able to incorporate information available in the unlabeled protein subcellular localization data, and hence, produce more robust classifiers than MMs and EM-MMs, when the fraction of labeled protein subcellular localization data is small.

Again as can be seen in Figure 2, AAMM(l+u) is superior in performance to MM, especially when small amounts of labeled data are available. For example, on plant, with 75 labeled sequences (i.e., 10% of labeled data), MM achieves 39% accuracy as compared to 44% obtained using AAMM(l+u) (|*t*| = 3.07). On **non-plant**, with 219 labeled sequences (i.e., 10% of labeled data), MM achieves 51% accuracy whereas AAMM(l+u) achieves 64% (|*t*| = 14). AAMM(l+u) not only incorporates information available in the unlabeled data (see previous comparison), but also performs parameter smoothing. Thus, AAMM(l+u) provides more robust estimates of model parameters than MMs, and hence, help reduce *overfitting* when the labeled training set is limited in size.

Both AAMM(l+u) and EM-MM make use of information available in the unlabeled data (i.e., both improve the performance of their counterpart classifiers trained only from labeled data) on all three data sets, although the improvement is not very large on **psortNeg** (Figure 2). However, AAMM(l+u) uses the joint distribution over amino acids (independent of the class variable) to learn a more robust abstraction hierarchy (i.e., a finer partitioning of the set of *k*-grams), especially when the amount of labeled data is small, so that better estimates of parameters can be obtained. On the other hand, EM-MM uses the joint distribution over amino acids after an initial classifier has made predictions on the unlabeled data. When small amounts of labeled data are available, the predictions made by the initial classifier may not be reliable.

AAMM(l+u) significantly outperforms EM-MMs on **non-plant, plant**, and **psortNeg** data sets, when the fraction of labeled data is small (see Figure 2). For example, with only 22 labeled sequences on **non-plant** (i.e., 1% of labeled data), AAMM(l+u) achieves 59% accuracy while EM-MM achieves 42%, which gives 29% reduction in classification error (|*t*| = 8.83). Similarly, with only 8 labeled sequences on plant (i.e., 1% of labeled data), AAMM(l+u) achieves 34% accuracy as compared to 28% obtained by EM-MM, which gives 8% reduction in classification error (|*t*| = 4.66). As the amount of labeled data increases, EM-MM significantly outperforms AAMM(l+u). For example, with 767 labeled sequences on **non-plant** (i.e., 35% of labeled data), EM-MM achieves 69% accuracy while AAMM(l+u) achieves 67% (|*t*| = 4.87).

Note that EM may decrease rather than increase the accuracy of classifiers if the generative model assumptions are not satisfied (see Figure 2** plant** data set). A weighted EM (i.e., weighting *unlabeled* sequences less) [12] helped improved the performance of EM-MMs (data not shown). A similar approach could be considered in AAMMs during learning the abstraction hierarchies.

**Figure 3:**
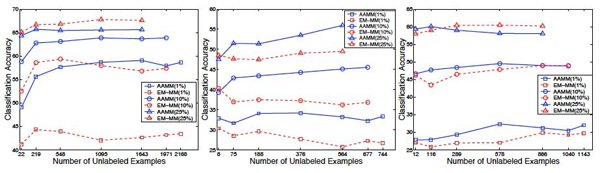
**Comparison of AAMMs with EM-MMs.** Comparison of AAMMs with EM-MMs for three different fractions of labeled data (i.e., 1%, 10%, and 25%) while varying the amount of unlabeled data on **non-plant** (left), **plant** (center), and **psortNeg** (right) data sets. *x* axis indicates the number of unlabeled examples in each data set corresponding to fractions of 1%, 10%, 25%, 50%, 75%, 90%, 99% of training data being treated as unlabeled data.

Figure 3 shows results of comparison of AAMMs with EM-MMs on **non-plant, plant**, and **psortNeg** data sets, respectively, while varying the amount of unlabeled data for three different fractions of labeled data (i.e., 1%, 10%, and 25% of labeled data) that are kept fixed. The *x* axis indicates the number of unlabeled examples in each data set.

As can be seen in Figure 3, the improvement in performance of AAMMs over EM-MMs is rather dramatic when the amount of labeled data is quite small. For example, when only 1% of labeled data is used regardless of the amount of unlabeled data, AAMMs consistently significantly outperform EM-MMs on **non-plant** and **plant** data sets (the largest and smallest *t* values on **non-plant** are 10.96 and 5.66, respectively). However, the difference in performance between AAMMs and EM-MMs diminishes as more and more labeled data become available (and eventually levels off). When the amount of labeled data is increased (e.g., 25% of labeled data), EM-MMs often significantly outperform AAMMs (Figures 3(a) and 3(c)). For example, on **non-plant** with 25% of unlabeled data, EM-MM achieves 68% accuracy, whereas AAMM achieves 66% (|*t*| = 7).

The classification accuracy of AAMMs typically increases with the amount of unlabeled data (when the subset of labeled data is fixed) (see Figure 3). For example, on **non-plant**, AAMM with 22 labeled sequences (i.e., 1% of labeled data) and 219 unlabeled sequences (i.e., 10% of unlabeled data) achieves an accuracy of 56% as compared to 49% obtained by AAMM with 22 labeled sequences (i.e., 1% of labeled data) and 22 unlabeled sequences (i.e., 1% of unlabeled data), 14% reduction in classification error.

AAMMs are comparable in performance with, and in some cases outperform, the co-training procedure, which uses MMs trained on different views of the protein subcellular localization data.

**Figure 4:**
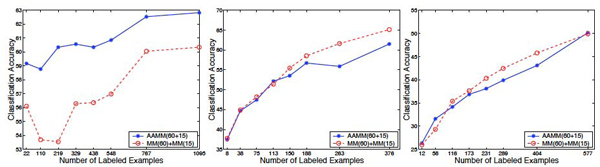
**Comparison of AAMMs with co-training MMs**. Comparison of AAMMs with co-training MMs on **non-plant** (left), **plant** (center), and **psortNeg** (right) data sets. AAMMs are trained on the first 60 and the last 15 amino acids of each protein sequence, AAMM(60 + 15). Co-training MMs consists of two co-trained MMs, one trained on the first 60 amino acids of each sequence, the other trained on the last 15 amino acids of each sequence. *x* axis indicates the number of labeled examples in each data set corresponding to fractions of 1%, 5%, 10%, 15%, 20%, 25%, 35%, 50% of training data being treated as labeled data. The fraction of unlabeled data in each data set is fixed to 50%.

Figure 4 shows results of experiments that compare AAMMs with co-training MMs on **non-plant**, **plant**, and **psortNeg** data sets, where we fixed the number of unlabeled examples (to 50%) and varied the number of labeled examples (from 1% to 50% as before). The x axis indicates the number of labeled examples in each data set. The number of unlabeled examples is kept fixed and is equal to the rightmost number of labeled examples on the *x* axis of each plot.

As can be seen in the figure, AAMMs trained on the first 60 and last 15 amino acids of each protein sequence significantly outperform two co-trained MMs, one trained on the first 60 amino acids of each sequence, and the other trained on the last 15 amino acids of each sequence on the **non-plant** data set. For example, with 22 labeled sequences (i.e., 1% of labeled data), AAMM achieves 59% accuracy while co-training MMs achieves 56% (|*t*| = 7.14). With 548 labeled sequences (i.e., 25% of labeled data), the accuracy of AAMM is 61%, whereas that of co-training MMs is 57% (|*t*| = 7.12). These results give 1% reduction in classification error .

However, on **plant** and **psortNeg** data sets, AAMMs are comparable in performance with co-training MMs. For example, on **plant** data set using 188 labeled sequences (i.e., 25% of labeled data), the accuracy of AAMM is 57%, whereas that of co-training MMs is 58% (the null hypothesis is not rejected, |*t*| = 0.61).

## Summary and discussion

Identifying subcellular localization of proteins is an important problem with broad applications in computational biology, e.g., rational drug design. Computational tools for identifying protein subcellular localization that can exploit large amounts of unlabeled data together with limited amounts of labeled data are especially important because of the high cost and efforts involved in labeling the data.

In this study, we presented an *abstraction-based* approach to *semi-supervised* learning of classifiers for the protein subcellular localization prediction task. Our approach utilizes abstraction augmented Markov models [25], which extend higher order Markov models by adding new variables corresponding to abstractions of *k*-grams (i.e., substrings of a fixed length *k*). AAMMs are probabilistic generative models that have the ability to incorporate information available in the *unlabeled* data: initially, an abstraction hierarchy over the k-grams is constructed from both labeled and unlabeled data, independent of the class variable. The labeled data is used to estimate the model parameters, based on the resulting abstraction hierarchy.

In this paper we compare AAMMs with MMs and EM-MMs and co-trained MMs. The results of our experiments on the subcellular localization prediction task show that semi-supervised AAMMs: (i) can effectively exploit unlabeled data; (ii) are more accurate than both the MMs and the EM based semi-supervised MMs; and (iii) are comparable in performance, and in some cases outperform, the co-training based semi-supervised MMs.

### Related work on semi-supervised learning

A variety of approaches to semi-supervised learning have been studied in the literature (see [11], [10] for reviews). Most of the existing semi-supervised learning algorithms including those based on co-training [13], Expectation Maximization (EM) [12], Transductive Support Vector Machines (TSVM) [14], cluster kernel [28], manifold based approaches [29,30], essentially involve different means of transferring labels from* labeled* to* unlabeled* samples in the process of learning a classifier that can generalize well on new unseen data.

EM-based methods provide a way to estimate the parameters of a generative model from* incomplete* data [26], i.e., samples that contain missing values for some of the variables. Semi-supervised learning is a special case of such inference where it is the class labels that are missing for a subset of the data [12]. Specifically, the parameters of the model are estimated initially from the labeled fraction of the training data, *D_L_*, and the resulting model is used to predict *p*(*y*|x) for each of the unlabeled samples in *D_U_*. The parameters are re-estimated using the entire training data *D* and this process is repeated until the estimates converge. Co-training [13] is a variant of this approach where unlabeled data are labeled with two different classifiers trained on different subsets of the features in x.

Several semi-supervised learning algorithms based on discriminative approaches to classification have been investigated. TSVM [14] can be seen as a discriminative counterpart of EM. TSVM starts by training an SVM on the labeled data and uses the trained SVM to label the unlabeled data. The algorithm iteratively attempts to maximize the margin of separation between the sets of samples labeled by the SVM (by considering at each iteration, alternative labels for pairs of originally unlabeled samples that have been assigned different labels by the SVM). A similar outcome can be achieved by adding an additional regularization term for unlabeled data to the objective function optimized by SVM [10]. Similar approaches for exploiting unlabeled data in training discriminative classifiers include [31], [32], [33], [34].

An alternative approach to exploiting unlabeled data relies on the manifold assumption: high-dimensional data lies on a lower dimensional manifold, making it possible to propagate labels from labeled samples to unlabeled samples based on some measure of closeness of the data points on the manifold. The manifold can be approximated by a weighted graph in which the nodes correspond to data samples and the weights on the links between nodes correspond to the pairwise similarity of the corresponding data points [35]. A number of techniques for label propagation have been proposed [29], [30]. Note that graph laplacian based techniques can be interpreted as a more general type of regularization where not only the *L*2 norm of the hypothesis is penalized but also the *L*2 norm of the hypothesis gradient.

In contrast to the approaches reviewed above, we present a novel *abstraction-based* approach to semi-supervised learning of sequence classifiers. We compared the semi-supervised AAMMs with the semi-supervised variants of Markov models trained using expectation maximization [12], and using co-training [13], [10].

### Expectation Maximization applied to Markov models

EM applied to MMs (EM-MMs) involves an iterative process of E- and M-steps. Specifically, an initial Markov model is learned only from *labeled* sequences *D_L_* using Equations (3), (4), and (5) (initialization step). The current model is used to assign *probabilistic* labels to the (originally) *unlabeled* sequences *D_U_* (i.e., to calculate the probability that each class generated an unlabeled sequence, , *u* =1,…,|*D_U_*|) using Equation (6) (E-step). Next, a new model is learned from originally labeled sequences  combined with the newly probabilistically labeled sequences , which were originally unlabeled, using Equations (3), (4), and (5) (M-step) (See Methods section for Equations (3), (4), (5), and (6) for details). E- and M- steps are repeated until the model does not change from one iteration to another [12].

### Co-training of Markov models

Let *D_L_* be a set of labeled examples, and *D_U_* a set of unlabeled examples. A set *D_U′_* is obtained by sampling *u* examples from *D_U_* (we used *u* = 75 examples in experiments). Each example x has two views, i.e., can be encoded with two different sets of features, x^(1)^ and x^(2)^. First, use *D_L_* and the x^(1)^ encoding to train a classifier *h*_1_, and *D_L_* and the x^(2)^ encoding to train another classifier *h*_2_. Second, classify the examples in *D_U′_* using *h*_1_ and *h*_2_ separately. Select *h*_1_’s and *h*_2_’s top (*k_j_*)*_j=_*_1,_…,|*C*| most confident predictions from each class (corresponding to the underlying data distribution), add them to *D_L_*, and remove them from *D_U′_*. Sample 2  examples from *D_U_* and move them to *D_U′_*. This process is repeated for a fixed number of iterations, or until all unlabeled data are used up [13], [10] (in experiments, we iterated until all unlabeled data was used). In co-training, the idea is that the two classifiers *teach one another* by re-training each classifier on the data enriched with predicted examples that the other classifier is most confident about.

### Semi-supervised abstraction augmented Markov models - our approach

Our abstraction-based approach to learning classifiers for the protein subcellular localization prediction task exploits large amounts of *unlabeled* data together with small amounts of *labeled* data to construct more robust abstraction hierarchies over the values of the *parents* of each node in a Markov model. Two values (*k*-grams) are clustered together if they induce similar conditional distributions of the next node, independent of the class. When the data are scarce, the estimates of joint probabilities are not reliable. However, the unlabeled data contain information about the joint probability distribution over sequence elements, and can help improve the statistical estimates of parameters. The abstraction hierarchy is subsequently used to learn a Markov model with abstract values of the parents.

It is worth mentioning that part of the AAMM is the representation of the clustering. Specifically, after the abstraction hierarchy is learned, for a given choice of the size *m* of an *m*-cut that defines an AAMM, an array of indices of size equal to the number of unique *k*-grams specifies the mapping between *k*-grams and abstractions (the space complexity is |*X*|*^k^*, where *X* is the alphabet). However, the number of parameters of AAMM (for a given class) based on such an abstraction hierarchy and an *m*-cut is *m*|*X*|, as opposed to |*X*|*^k^*|*X*| in the case of MMs, where *m* ≪ |*X*|*^k^*.

While AAMMs reduce the complexity of the learned model, some information is lost due to *abstraction.* It is of interest to incorporate into AAMMs some means of gracefully trading off the complexity of the model against its predictive accuracy. One way to do this is to augment the algorithm, e.g., by designing an MDL-based scoring function to guide a top-down search for an optimal cut [36].

AAMMs not only significantly outperform MMs but also are simpler than MMs, and hence easier to interpret from a biological standpoint: the set of k-grams in an abstraction can be seen as a sequence profile (e.g., Position Specific Scoring Matrix).

The results of our experiments show that AAMMs can make effective use of *unlabeled* data and that AAMMs significantly outperform EM-MMs when the amount of *labeled* data is very small, and relatively large amounts of *unlabeled* data are readily available. Here, because of the small amounts of *labeled* data available, the ability of AAMMs to minimize overfitting (through parameter smoothing) turns out to be especially beneficial. In comparing semi-supervised AAMMs with the previous semi-supervised work on the protein subcellular localization prediction task, we found that AAMMs are competitive with, and in some cases outperform, co-training of MMs.

The results presented here demonstrate the effectiveness of an abstraction-based approach to exploiting unlabeled data in a semi-supervised setting on the protein subcellular localization prediction task. Such an approach can in principle be combined with existing semi-supervised learning techniques including those that use EM, co-training, manifold assumption (propagation of labels from labeled to unlabeled samples based on some similarity measure between samples).

Our current implementation of AAMM constructs an abstraction hierarchy over the values of the *k* predecessors of a sequence element by grouping them together if they induce similar conditional distributions over that element of the sequence. It would be interesting to explore alternative approaches to building abstraction hierarchies, e.g., probabilistic suffix trees (PSTs) [37].

## Methods

In this section, we briefly described the data sets used in experiments, provide some background on Markov models for sequence classification, and then present our novel AAMM-based approach to semi-supervised learning.

### Data sets

The first and second data sets used in our experiments, **plant** and **non-plant **[38], were first introduced in [4]. The plant data set contains 940 examples belonging to one of the following four classes: *chloroplast* (141),* mitochondrial* (368), *secretory pathway/signal peptide* (269) and *other* (consisting of 54 examples with label nuclear and 108 examples with label cytosolic). The **non-plant** data set contains 2738 examples, each in one of the following three classes: *mitochondrial* (361), *secretory pathway/signal peptide* (715) and *other* (consisting of 1214 examples labeled nuclear and 438 examples labeled cytosolic).

The third data set used in our experiments, PSORTdb v.2.0 [39] Gram-negative sequences, introduced in [40], contains experimentally verified localization sites. We refer to this data set as **psortNeg**. We use all proteins that belong to exactly one of the following five classes: *cytoplasm* (278), *cytoplasmic membrane* (309), *periplasm* (276), *outer membrane* (391) and *extracellular* (190). The total number of examples (proteins) in this data set is 1444.

### Markov models

Markov models (MMs) are probabilistic generative models that assume a mixture model as the underlying model that generated the sequence data. Each mixture component corresponds to a class *c_j_* ∈ *C* = {*c*_1_,…,*c*_|_*_c_*_|_}. A sequence is generated according to a set of parameters, denoted by *θ*, that define the model.

**Figure 5:**
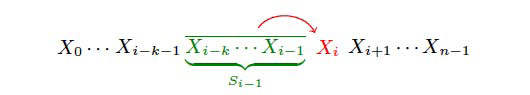
**Markov model for sequence classification**. Dependency of *X_i_* on *X_i_*_−_*_k_*,…,*X_i_*_−1_ in a *k^th^* order Markov model.

Let x = *x*_0_…*x_n_*_−1_ be a sequence over a finite alphabet *X*, x ∈ X*, and let y denote x’s class (note that if x was generated by the *j^th^* mixture component, then *y* = *c_j_*). Let *X_i_*, for *i* = 0,…, *n* − 1, denote the random variables corresponding to the sequence elements *x_i_* in x. In a *k^th^* order MM, the sequence elements satisfy the *Markov property*: . That is, *X_i_* is conditionally independent of *X*_0_,…, *X_i_*_−_*_k_*_−1_ given *X_i_*_−_*_k_*,…, *X_i_*_−1_ for *i* = *k*,…, *n* − 1. *X_i_*_−_*_k_*,…, *X_i_*_−1_ are called *parents* of *X_i_*. Figure 5 shows the dependency of *X_i_* on *X_i−k_*,…,*X_i_*_−1_ in a *k^th^* order MM. Hence,

*p*(*x_i_*|*x*_0_…*x_i_*_−1_,*c_j_*;*θ*) = *p*(*x_i_*|*x_i_*_−_*_k_…x_i_*_−1_,*c_j_*;*θ*). (1)

The probability of x given its class *c_j_*, *p*(x|*c_j_*;θ), can be written as follows:

 (2)

Let *S_i_*_−1_ denote the* parents X_j_*_−_*_k_*…*X_i_*_−1_ of *X_i_*. The values of *S_i_*_−1_ represent instantiations of *X_i_*_−_*_k_*…*X_i_*_−1_, which are substrings of length *k* (i.e., *k*-grams) over the alphabet *X*. Let* S* denote the set of *k*-grams over *X*, s denote a *k*-gram in *S*, and *σ* a symbol in *X*. The cardinality of *S* is |*X*|*^k^* and is denoted by *N*.

The set of parameters *θ* of an MM is: , where , and .

### Learning Markov models

Given a* labeled* training set , learning a Markov model reduces to estimating the set of parameters *θ* from *D_L_*, using the maximum likelihood estimation [41]. The estimate  of  is obtained from *D_L_* as follows:

  (3)

where #[*sσ*, x*_l_*] is the number of times the symbol *σ* “follows” the *k*-gram *s* in the sequence x*_l_*, and *p*(*y_l_* =* c_j_*|x*_l_*) ∈ {0,1} is obtained based on the sequence label.

The estimate  of  is obtained from *D_L_* as follows:

 (4)

where #[*s*, x*_l_*] is the number of times *s* occurs in x*_l_*.

The class prior probabilities  are estimated as follows:

 (5)

We used Laplace correction to obtain smoothed estimates.

### Using Markov models for classification

Classification of a new sequence x requires computation of conditional probability . Applying Bayes rule:

 (6)

The class with the highest posterior probability,  is assigned to x.

## Semi-supervised AAMM

We first provide the AAMM definitions and then describe how to learn semi-supervised AAMMs.

### AAMMs

AAMMs effectively reduce the number of parameters of a *k^th^* order MM (which is exponential in *k*) by learning an abstraction hierarchy (AH) over the set of *k*-grams *S*.

**Definition 1 (Abstraction Hierarchy)***An abstraction hierarchy T over a set of k-grams S is a rooted tree such that: (1) the root of T denotes S; (2) the leaves of T correspond to singleton sets containing individual k-grams in S; (3) the children of each internal node (say* a*) correspond to a partition of the set of k-grams denoted by* a. *Thus,* a *denotes an abstraction or grouping of “similar” k-grams.*

**Figure 6:**
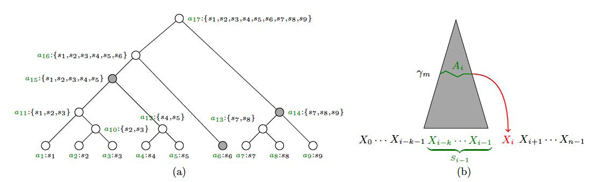
**Abstraction augmented Markov models**. (a) An abstraction hierarchy *T* on a set *S* = {*s*_1_,…,*s*_9_} of 2-grams over an alphabet of size 3. The abstractions *a*_1_ to *a*_9_ correspond to the 2-grams *s*_1_ to *s*_9_, respectively. The subset of nodes *A* = {*a*_15_, *a*_6_, *a*_14_} represents a 3-cut γ_3_ through *T*; (b) Dependency of *X_i_* on *A_i_*, which takes values in a set of abstractions *A* corresponding to an *m*-cut γ*_m_*, in a *k_th_* order AAMM.

Note that each internal node (or *abstraction* a) contains the subset of *k*-grams at the leaves of the subtree rooted at a. Figure 6(a) shows an example of an AH *T* on a set *S* = {*s*_1_,…,*s*_9_} of 2-grams over an alphabet of size 3.

**Definition 2 (m-Cut)***An m-cut* γ*_m_ through an abstraction hierarchy T is a subset of m nodes of T such that for any leaf s* ∈ *S, either s* ∈ γ*_m_ or s is a descendant of some node in* γ*_m_. The set of abstractions A at any given m-cut* γ*_m_ forms a partition of S.*

Specifically, an *m*-cut γ*_m_* partitions the set *S* of *k*-grams into *m* (*m* ≤ *N* = |*S*|) non-overlapping subsets *A* = {*a*_1_ : *S*_1_,…,*a_m_*:*S_m_*}, where *a_i_* denotes the *i*-th abstraction and *S_i_* denotes the subset of *k*-grams that are grouped together into the *i*-th abstraction based on some similarity measure. Note that *S*_1_ ∪…∪ *S_m_* = *S* and ∀1 ≤ *i*, *j* ≤ *m*, *S_i_ ∩ S_j_ =* ∅. In Figure 6(a), the subset of nodes {*a*_15_, *a*_6_, *a*_14_} represents a 3-cut γ_3_ through *T*.

AAMMs extend the graphical structure of MMs by introducing new variables *A_i_* that represent abstractions over the values of *S_i_*_−1_, for *i* = *k*,…, *n* − 1 (Figure 6(b)). Each *A_i_* takes values in the set of abstractions *A* = {*a*_1_,…,*a_m_*} corresponding to an *m*-cut, γ*_m_*. We model the fact that *A_i_* is an abstraction of *S_i_*_−1_ by defining *p*(*A_i_* = *a_i_*|*S_i_*_−1_ = *s_i_*_−1_) = 1 if *s_i_*_−1_ ∈ *a_i_*, and 0 otherwise, where *s_i_*_−1_ ∈ *S* and *a_i_* ∈ *A* represent instantiations of variables *S_i_*_−1_ and *A_i_*, respectively. Furthermore, in AAMMs, the node *X_i_* directly depends on *A_i_* instead of being directly dependent on* S_i_*_−1_, as in the standard MMs. Hence, the probability of x given its class, *p*(x|*c_j_*;*θ*), can be written as follows:

 (7)

The set of parameters* θ* of an AAMM is: , where , and .

### Learning semi-supervised AAMMs

In what follows we show how to learn AAMMs from both *labeled* and *unlabeled* data. This involves: learning abstraction hierarchies from both *labeled* and *unlabeled* data; and learning model parameters from *labeled* data using the resulting abstraction hierarchy.

#### *Learning abstraction hierarchies*

The algorithm for learning AHs over a set *S* of *k*-grams starts by initializing the set of abstractions *A* such that each abstraction *a_j_* ∈ *A* corresponds to a *k*-gram* s_j_* ∈ *S*, *j* = 1,…,*N*. The leaves of the AH *T* are initialized with elements of *S*. The algorithm recursively merges pairs of abstractions that are most “similar” to each other and terminates with an abstraction hierarchy after *N* − 1 steps. We store *T* in a last-in-first-out (LIFO) stack. For a given choice of the size *m* of an *m*-cut through *T*, the set of abstractions that define an AAMM can be extracted by discarding *m* − 1 elements from the top of the stack.

We consider two *k*-grams to be “similar” if *they occur within similar contexts.* In our case, we define the *context of a k-gram s* ∈ *S* to be the conditional probability distribution of the next letter in the sequence given the *k*-gram, *p*(*X_i_*|*s*), independent of the class variable. Hence, this can be estimated from both *labeled* sequences *D_L_* and *unlabeled* sequences *D_U_* as follows:

  (8)

where #[*sσ*, x*_l_*] and #[*sσ*, x*_u_*] represent the number of times the symbol *σ* “follows” the *k*-gram *s* in the sequence x*_l_*, and x*_u_*, respectively.

Since an abstraction is a set of *k*-grams, the* context of an abstraction a* = {*s*_1_,…, *s*_|_*_a_*_|_} is obtained by a weighted aggregation of the contexts of its *k*-grams. That is,

  (9)

where .

We identify the most “similar” abstractions as those that have the smallest weighted Jensen-Shannon divergence between their contexts. JS divergence [42] provides a natural way to compute the distance between two probability distributions that represent contexts of two abstractions. Specifically, we define the distance between two abstractions *a′* and *a″* in *D* as follows:

where  and .

#### *Learning AAMM parameters*

Given a* labeled* training set , learning an AAMM reduces to estimating the set of parameters *θ* from *D_L_*, denoted by . This can be done as follows: use Equation (3) to obtain the estimates  of  for any *k*-gram *s* ∈ *S* (note that these estimates correspond to the estimates  when *a* = {*s*}, i.e., the leaf level in the AH *T*). The estimates  of ,  when *a* = {*s*_1_,…,*s*_|_*_a_*_|_}, are a weighted aggregation of the estimates of *a*’s constituent *k*-grams, i.e.,

 (10)

where . Use Equations (4) and (5) to obtain the estimates  of  and  of , respectively.

#### *Using AAMMs for classification*

Given a new sequence x = *x*_0_,…,*x_n_*_−1_ and an *m*-cut γ*_m_* through *T*,  can be computed as follows: initialize  by  ; parse the sequence from left to right. For each *k*-gram *x_i_*_−_*_k_*…*x_i_*_−1_ find the abstraction* a_w_* ∈ γ*_m_* it belongs to and retrieve the parameters associated with* a_w_*. Successively multiply  for *i* = *k*,…, *n* − 1 to obtain .

As in MMs, apply Bayes rule to obtain  and assign the class with the highest posterior probability to x.

## Competing interests

The authors declare that they have no competing interests.

## Authors’ contributions

All authors read and approved the final manuscript.
